# Use of Mesenchymal Stem Cell Transplantation as a Treatment for Liver Cirrhosis in Animal Models

**DOI:** 10.7759/cureus.71720

**Published:** 2024-10-17

**Authors:** Alexander G Skuratov, Boris B Osipov, Anatoly N Lyzikov, Dmitry A Zinovkin, Imran M Adam, Mark L Kaplan, Anton A Prisentsov, Evgeny V Voropaev, Madusha M P Angage

**Affiliations:** 1 Department of Surgical Diseases No1 with the Course of Cardiovascular Surgery, Gomel State Medical University, Gomel, BLR; 2 Department of Pathology, Gomel State Medical University, Gomel, BLR; 3 Department of Research, Gomel State Medical University, Gomel, BLR

**Keywords:** experimental animal study, liver cirrhosis. fibrosis, mesenchymal stem cells (mscs), stem cell therapy effectiveness, treatment methods

## Abstract

Introduction: Liver cirrhosis is one of the top 10 causes of death worldwide, and liver disease is in the top five in many developing countries. The treatment of liver cirrhosis at various stages necessitates the development of new organ transplantation techniques. One of these options is cell therapy, which has lately been used to treat a wide range of illnesses.

Methods: The study used a rabbit model of liver cirrhosis to examine the efficiency of mesenchymal stem cell (MSC) transplantation. Subcutaneous injections of carbon tetrachloride (CCL4) were used to cause liver cirrhosis. When liver cirrhosis developed, autologous mesenchymal stem cells were infused into the portal vein.

Results: The histological image of the cirrhotic liver improved one month after MSC transplantation.

Conclusion: This demonstrates that the intraportal delivery of autologous MSC to rabbits with experimental liver cirrhosis has good therapeutic outcomes.

## Introduction

Chronic, widespread liver problems are a serious and pressing issue in modern medicine and surgery. Millions of people worldwide suffer from liver disorders, which are a major cause of early disability and death. The major cause of portal hypertension and its complications is liver cirrhosis.

The incidence of liver cirrhosis was reported at 2.1 million cases in 2019, contributing to around 1.5 million fatalities worldwide [1]. Chronic liver disorders, including cirrhosis, are the 14th leading cause of mortality worldwide, accounting for a considerable number of deaths and disability-adjusted life years [2]. According to the Global Burden of Disease database, the incidence of liver cirrhosis and other chronic liver diseases in Belarus is 49,414 cases, with 1939 deaths [3].

Liver transplantation is the only established therapy for those suffering from end-stage liver disease. Nonetheless, despite enormous advances in transplantation over the past several decades, a number of concerns persist, including a scarcity of viable donor organs, high medical costs, and other factors that fall short of achieving the full need for liver transplantation. As a result, novel approaches to treating liver cirrhosis have become required in recent years. A global scientific investigation is now being undertaken on the use of cell therapy for the treatment of liver diseases [4-7]. The work's objective is to assess mesenchymal stem cell (MSC) transplantation's efficacy in treating experimental liver cirrhosis in rabbits.

## Materials and methods

To achieve this goal, we used experimental research methods on laboratory animals, as they allow us to give a comprehensive assessment and develop methods for adequate correction of liver failure, which is not always possible in clinical trials.

Among the toxic models, the model of liver damage induced by carbon tetrachloride (CCl_4_) has become widespread [8]. The administration of CCl_4 _to laboratory animals leads to early destruction of cytochrome P-450 liver microsomes, inhibition of the enzyme glucose-6-phosphatase, ultrastructurally detectable intense necrosis and fatty liver dystrophy, and eventually the development of liver cirrhosis.

White California rabbits were used as an object for modeling liver cirrhosis, which gave a number of advantages compared to small laboratory animals (rats, mice). Firstly, the ability of the rabbit liver to regenerate is lower than in rats, which brings the experimental conditions closer to real clinical conditions in humans. Secondly, there is the possibility not only of postmortem morphological examination of organs but also of lifetime morphological and functional examination (laboratory and instrumental) of pathological changes in "target organs" with lesser consequences for the animal [9].

**Figure 1:**
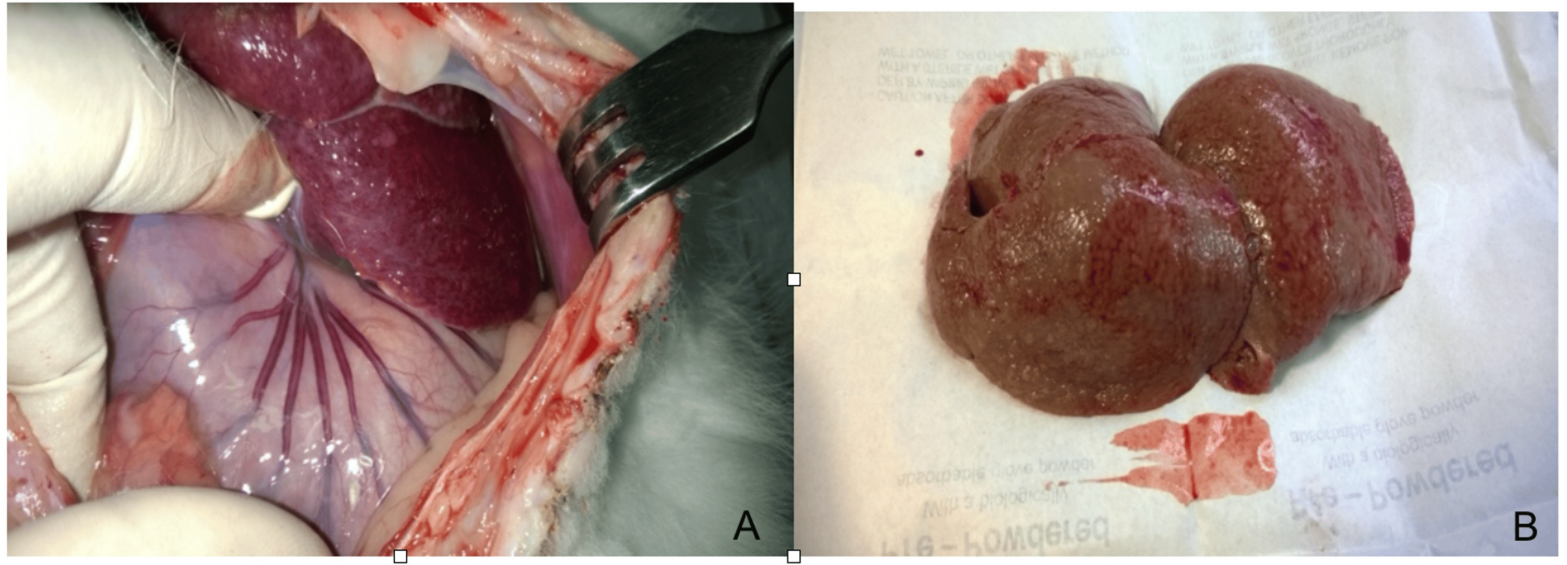
Characteristics of experimental liver cirrhosis after five months of carbon tetrachloride administration. A - photo of the rabbit's abdominal cavity. B – photo of rabbit liver.

Liver cirrhosis was simulated by subcutaneous injection of a 50% solution of CCl_4_ (carbon tetrachloride) in olive oil into a rabbit at a rate of 1 ml per kg of body weight twice a week for five months (Figure 1).

For the lifetime assessment of pathological changes developing during the experiment, laboratory methods (general blood analysis, biochemical blood tests, coagulogram) and instrumental (ultrasound, incision biopsy) diagnostics were used.

Ultrasound signs of liver cirrhosis and portal hypertension were studied: heterogeneous and increased echogenicity of the liver, changes in the size of the liver (anterior-posterior size), the diameter of the portal vein (vena portae, VP), and the presence of free fluid in the abdominal cavity. Ultrasound was performed on an Aloka SSD-500 (Tokyo, Japan) device.

Autologous MSCs were used as a therapeutic agent. The source of autologous MSCs was a section of fatty tissue in the inguinal region of a rabbit, which was collected from each rabbit under general anesthesia before the start of carbon tetrachloride injections. The isolation and cultivation of MSCs were carried out and then cultured in a CO_2_ incubator according to the standard protocol procedure [10]. The MSCs of the second and third passages were used for experiments. MSCs were typed according to their characteristic morphology and expression of marker genes (CD 90, 29, 44, 45, etc.). The injection of MSCs suspension into rabbits was performed under general anesthesia after upper median laparotomy by intraportal injection with an atraumatic (pencil point) needle G26. The concentration of MSCs in the suspension was 5x10^6^ in ml, the volume of the injected suspension was 3 ml, and the injection rate was 0.3 ml/sec. After removing the needle from the portal vein, if necessary, hemostasis was performed in the venipuncture area by pressing for three to five minutes. The wound was sutured tightly in layers.

The intact group (control group number one) consisted of three healthy rabbits that were not injected with carbon tetrachloride. They were kept in the same conditions as animals from other groups and were used to compare laboratory, instrumental, and histological parameters with animals from experimental groups.

Rabbits (N=20) with liver cirrhosis were divided into two groups: experimental group number two (control group with cirrhosis of the liver, N=10), without MSCs transplantation to study the natural pathogenesis of the disease; and experimental group number three (main group, N=10), with cell transplantation. Animals from both groups were kept in the same conditions in a vivarium.

On the day of carbon tetrachloride withdrawal, rabbits of experimental group number three underwent an upper median laparotomy, during which the condition of the liver and other organs was macroscopically assessed, an incisional liver biopsy was performed for histological examination, and a suspension of autologous MSCs was once injected into the portal vein according to the previously described method. The animals were removed from the experiment one month after MSC transplantation (day 30).

After removing the animals from the experiment, the pieces of organs were fixed in 10% neutral formalin and poured into paraffin blocks according to the standard procedure. The dewaxed sections of the liver were stained with hematoxylin-eosin and Van Gieson and Martius Scarlet Blue trichrome, after which the general morphological and morphometric picture of the organ was studied [11].

## Results

Characteristics of liver cirrhosis in rabbits on the day of carbon tetrachloride withdrawal

The administration of carbon tetrachloride to rabbits using the indicated approach resulted in the development of liver cirrhosis by the 20th week (end of the fifth month) of the experiment. Liver cirrhosis is post-necrotic and mostly multilobular. The macroscopic alterations in the liver and other organs, as well as the pathomorphological image of the resected portions of the liver on the day of carbon tetrachloride withdrawal, were comparable in rabbits from experimental groups two and three. Macroscopically, the liver was enlarged, light brown in hue, thick and rough, with a fine-grained structure in the section. Both the extra-hepatic and intra-hepatic bile ducts were dilated. There were additional symptoms of portal hypertension, including varicose veins in the stomach's cardiac region, splenomegaly, and ascites.

**Figure 2:**
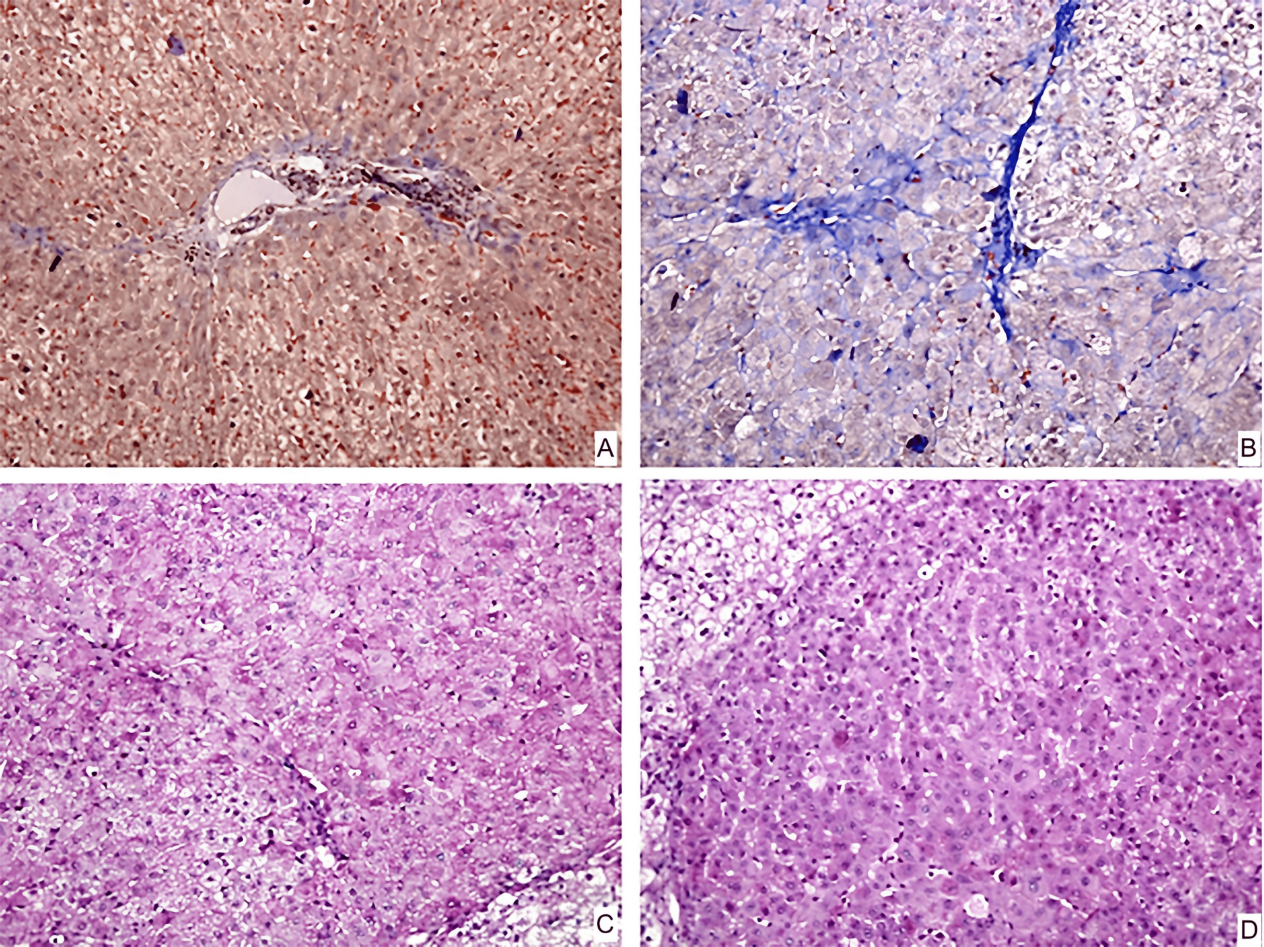
Morphological changes of the liver in rabbits of the first group. A – connective tissue septa at the beginning of the experiment. The color is trichrome by Martius Scarlet Blue. Magnification ×200; B – connective tissue septa at the end of the experiment. The color is trichrome by Martius Scarlet Blue. Magnification ×200; C – glycogen in hepatocytes at the beginning of the experiment. The coloring is PAS. Magnification ×200 D – glycogen in hepatocytes at the end of the experiment. The coloring is PAS. Magnification ×200 MSCs- Mesenchymal stem cells PAS- Periodic acid–Schiff

In group number one (control), microscopy on the day of carbon tetrachloride withdrawal revealed a liver of normal histological structure. Hepatic lobules are constructed from a system of hepatic plates converging towards the centre of the lobule and consisting of a single row of cells. There are single, double-core cells in the parenchyma around the vessels. In vessels and bile ducts of normal histological structure, the fibrous septa of lobules are thin (Figure 2A). The presence of glycogen in hepatocytes is noted (Figure 2B).

**Figure 3:**
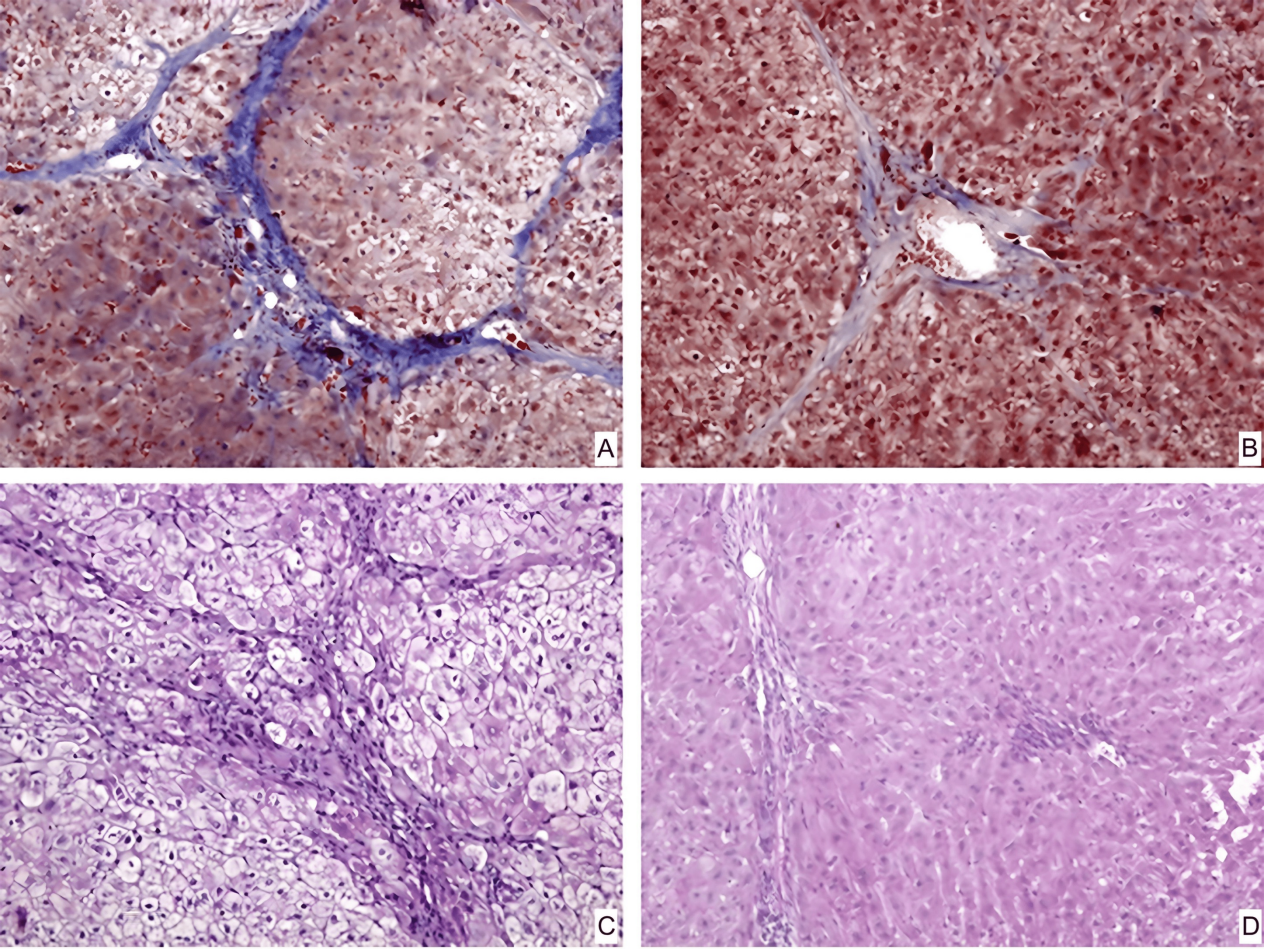
Pathomorphological changes of the liver in rabbits of the second group A – connective tissue septa at the beginning of the experiment. The color is trichrome by Martius Scarlet Blue. Magnification ×200; B – connective tissue septa at the end of the experiment. The color is trichrome by Martius Scarlet Blue. Magnification ×200; C – glycogen in hepatocytes at the beginning of the experiment. The coloring is PAS. Magnification ×200 D – glycogen in hepatocytes at the end of the experiment. The coloring is PAS. Magnification ×200 MSCs- Mesenchymal stem cells PAS- Periodic acid–Schiff

**Figure 4:**
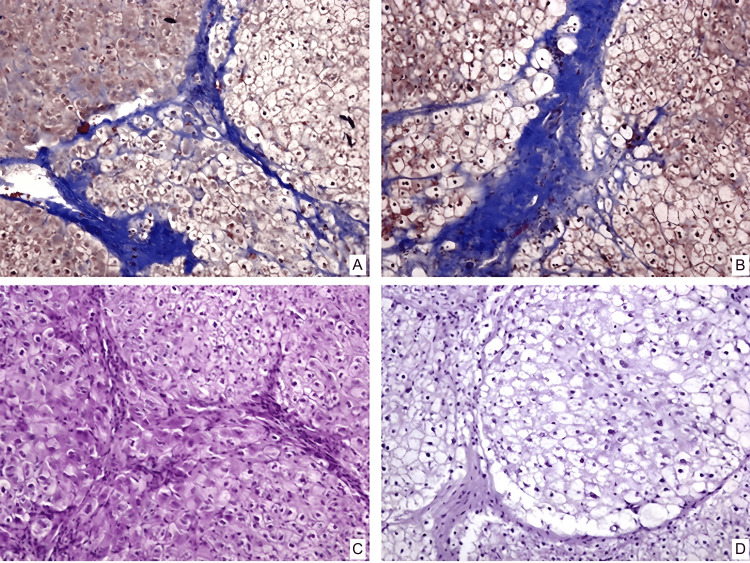
Pathomorphological changes of the liver in rabbits of the third group. A – connective tissue septa at the beginning of the experiment. The color is trichrome by Martius Scarlet Blue. Magnification ×200; B – connective tissue septa one month after MSC transplantation. The color is trichrome by Martius Scarlet Blue. Magnification ×200; C – glycogen in hepatocytes at the beginning of the experiment. The coloring is PAS. Magnification ×200; D – glycogen in hepatocytes one month after MSC transplantation. The coloring is PAS. Magnification ×200 MSCs- Mesenchymal stem cells PAS- Periodic acid–Schiff

In groups number two and number three, the histological picture is similar to each other, represented by the formation of coarse connective tissue septa with the formation of false lobules, areas of vascular proliferation, and bile capillaries in overgrown connective tissue (Figures 3A, 4A). Foci of weak lymphoid infiltration are detected. Hepatocytes have moderate to severe dystrophic changes and a decrease in glycogen in them (Figures 3C, 4C). There is a focal increase in the number of double-core cells.

**Figure 5:**
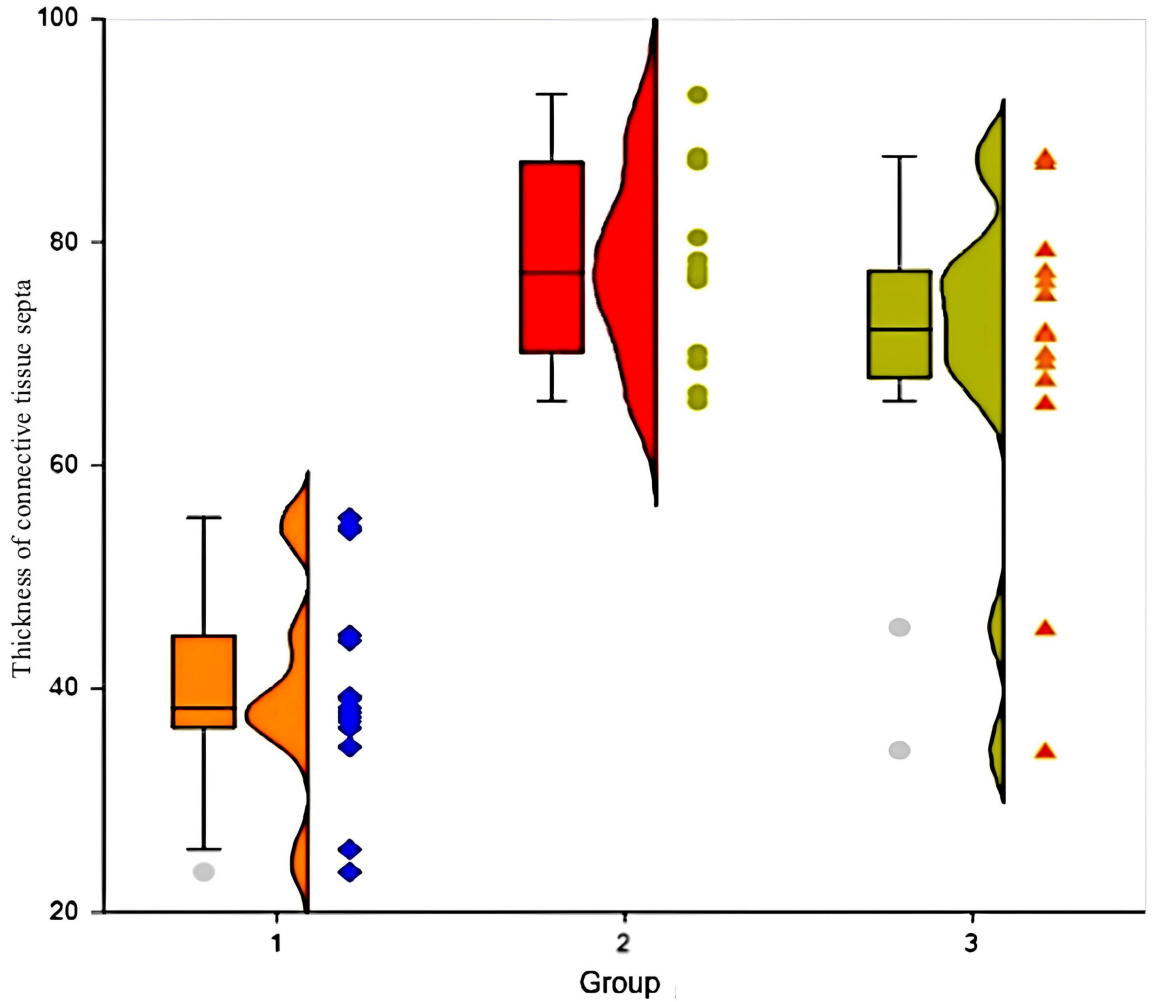
Thickness of connective tissue septa in rabbits of different groups on the day of carbon tetrachloride withdrawal

The thickness of the septa in group number one is 38.5 (37.3-44.8) microns, in group number two is 72.1 (67.8-77.5) microns, and in group number three is 77.3 (70.1-87.2) (Figure 5). In group one, the thickness of the septa is statistically lower than in group two (p = 0.0012, Mann-Whitney criterion) and group three (p=0.0008). There was no statistical difference in the thickness of the septa in groups two and three (p = 0.09). Dystrophic changes in hepatocytes in group one are not detected; in group two, they are detected in all cases; and in group three, these changes are observed in 86.7% of cases. When comparing group one with two and three, there is a statistically significant difference in both cases (p<0.001, Pearson chi-squared criterion). There was no statistical difference between groups two and three (p = 0.428).

**Figure 6:**
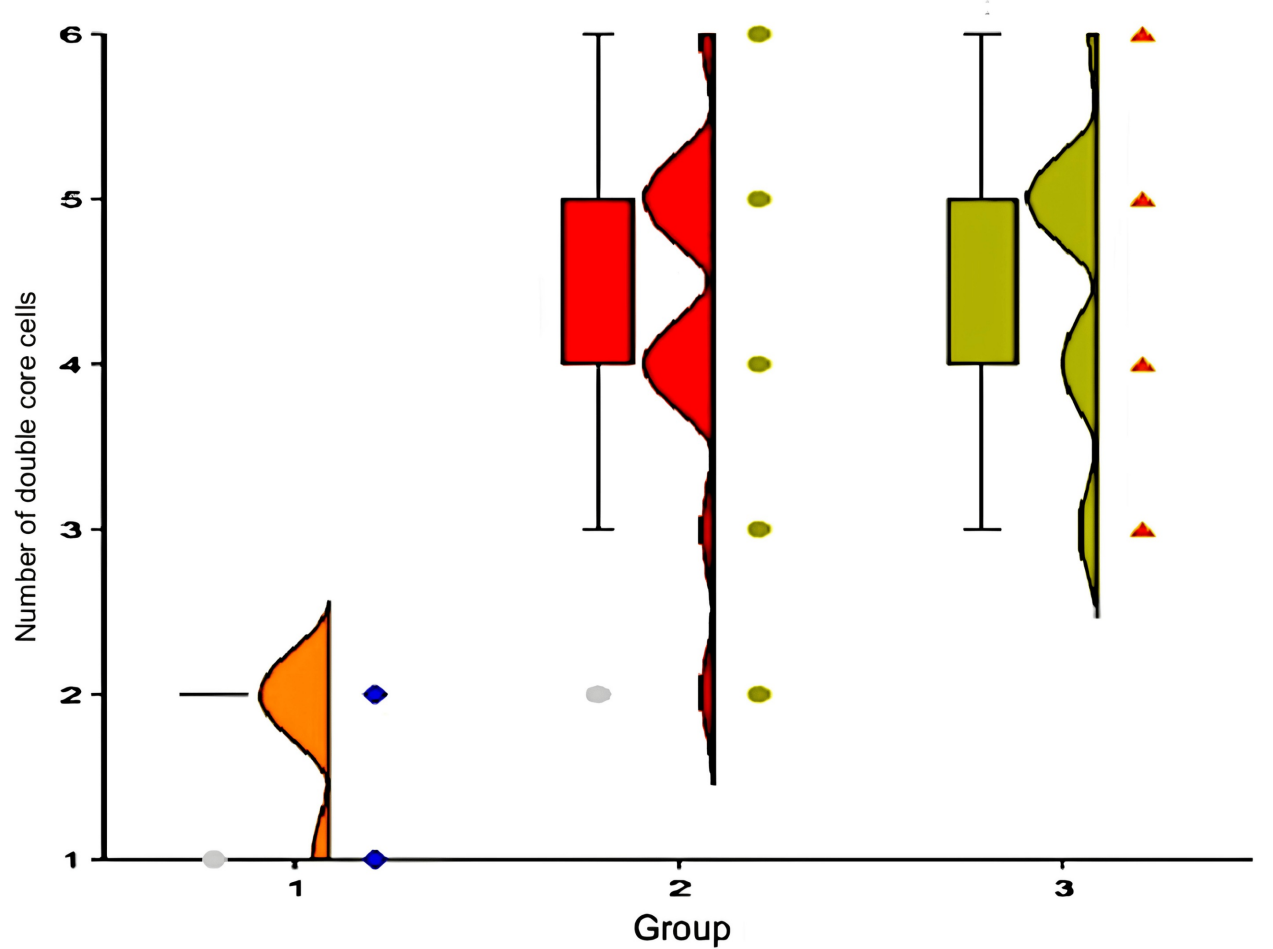
The number of double-core cells in rabbits of different groups on the day of carbon tetrachloride withdrawal

In group one, the number of double-core cells in the field of vision is two (2-2), in group two, four (4-5), and in group three, five (4-5). In group one, the number of double-core cells is statistically lower than in group two (p<0.01, Mann-Whitney criterion) and group three (p<0.01) (Figure 6). There was no statistical difference in the number of double-core cells in groups two and three (p = 0.604).

Characteristics of experimental liver cirrhosis in rabbits one month after transplantation of autologous MSCs

After developing liver cirrhosis, rabbits in group three were injected once with a suspension of autologous MSCs containing 5x10^6^ cells per kg of animal body weight into the portal vein. After one month, rabbits from all groups were withdrawn from the experiment to assess the efficacy of cell treatment for experimental cirrhosis of the liver. Microscopy in group one revealed the same image of the liver's normal histological structure as on the first day. The fullness of each vessel in the triads was calculated. Fibrous septa in hepatic lobules are thin (Figure 2B). The arteries and bile ducts had typical histological structure, and the fibrous septa between the lobules were thin. The lobule architecture remained intact, and the hepatocytes retained glycogen (Figure 2D). Single double-core cells were seen in the parenchyma around the arteries.

In group two, the histological image of liver cirrhosis was preserved. Microscopy revealed coarse connective tissue septa, which formed spurious lobules (Figure 3B). Vascular growth and bile capillaries were seen in enlarged connective tissue in the liver's peripheral sections. Isolated clumps of lymphoid cells were discovered in the stroma, primarily around the arteries. Hepatocytes exhibited substantial dystrophic alterations (Figure 3D), with a localized increase in two-core cells. All of this implies that the degenerative alterations in the liver continue after the removal of carbon tetrachloride.

In group three, damage to the liver's cellular architecture was identified due to fibrous growths and the existence of false lobules; nonetheless, there was a significant thinning of connective tissue (Figure 4B). Dystrophic alterations were only seen on the periphery of the hepatic lobules and were minimally pronounced. In the center parts of the hepatic lobules, glycogen in hepatocytes and perivascular zones of the typical lamellar structure of the hepatic lobule with a high glycogen content in cells was seen (Figure 4D).

**Figure 7:**
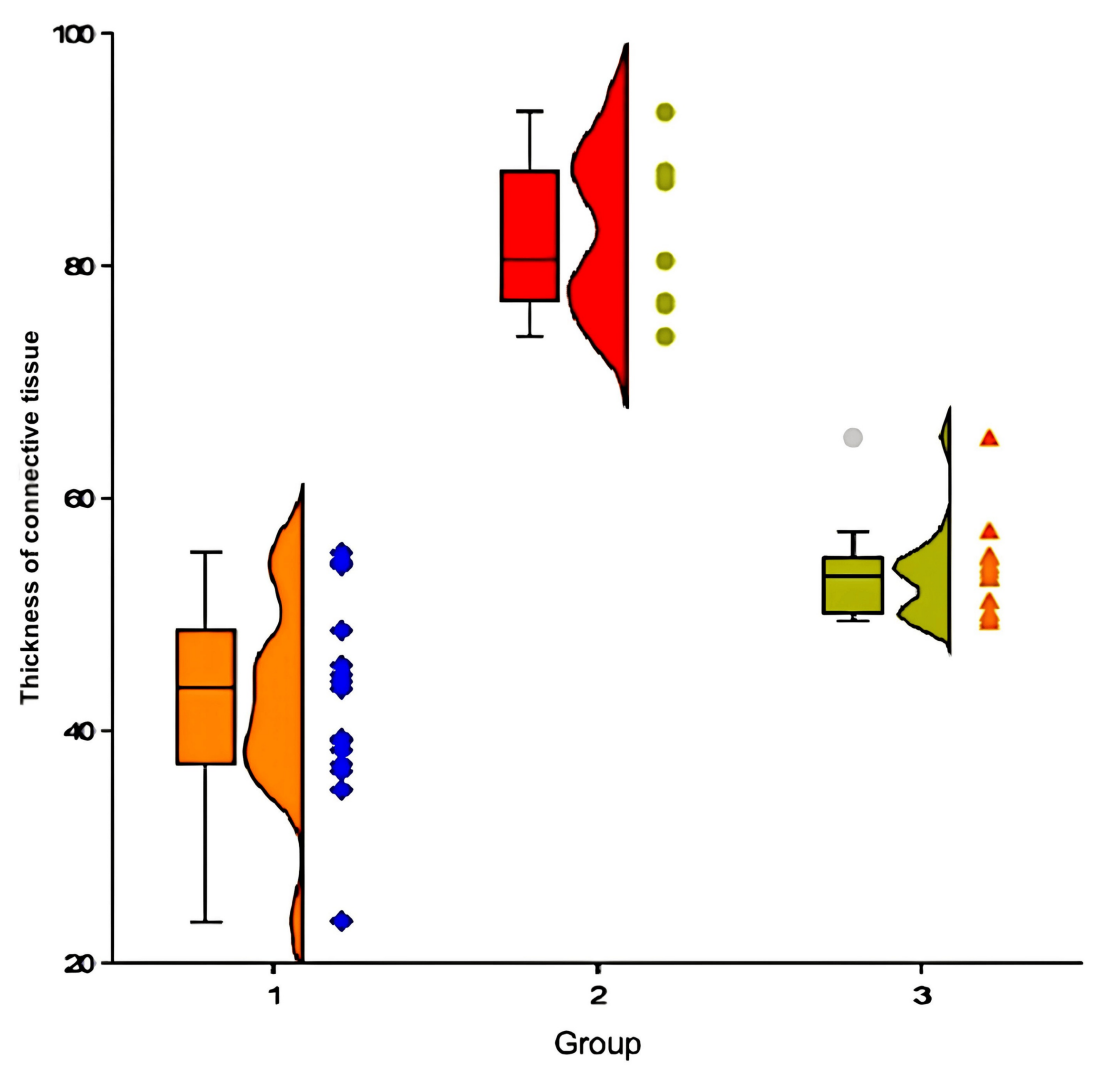
Thickness of connective tissue septa in rabbits of different groups one month after administration of MSCs MSCs- Mesenchymal stem cells

The septa thickness varied by group: 43.6 (23.6-55.2) microns in group one, 80.4 (73.9-90.1) microns in group two, and 53.3 (49.4-57.2) microns in group three (Figure 7). Group one had significantly thinner septa compared to groups two (p<0.01, Mann-Whitney criteria) and three (p<0.01), respectively. A significant difference in septum thickness was found between groups two and three (p<0.001).

Dystrophic alterations in hepatocytes were not discovered in group two; they were detected in 80% of instances; and in group three, they were seen in 20% of cases. A significant difference was seen between groups one and two (p<0.001, Pearson chi-squared criteria), but not between groups one and three (p=0.224). A significant difference was seen between groups two and three (p<0.001).

**Figure 8:**
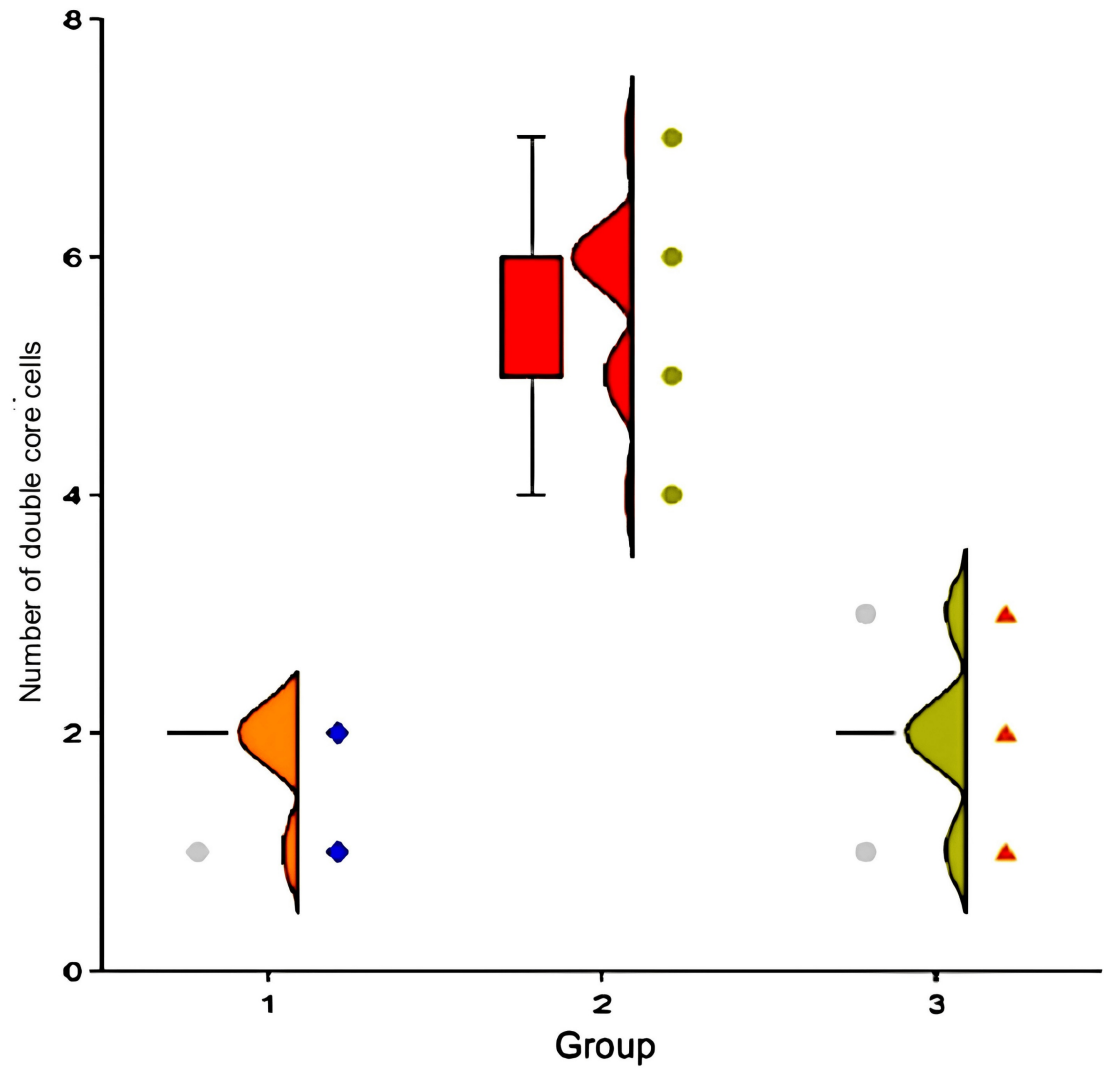
The number of double-core cells in rabbits of different groups one month after the introduction of MSCs MSCs- Mesenchymal stem cells

The median number of binuclear cells in the visual field was two (2-2) in group one, five (six in group two), and three (two in group three). Figure 8 depicts the groups' characteristics based on the number of double-core cells. The number of double-core cells was significantly lower in group one compared to group two (p<0.001, Mann-Whitney criteria), but not significantly different from group three (p = 0.46). A significant difference in the number of double-core cells was observed between groups two and three (p<0.001).

The following is an analysis of morphometric markers in each group individually. For clarity, we offer a summary of the average values (median, 25th and 75th percentiles) of the thickness of connective tissue septa in different groups before the introduction of MSC to rabbits in group three and one month following cell therapy (Table 1).

**Table 1:** Thickness of connective tissue septa in different groups before and after injection of MSCs MSCs- Mesenchymal stem cells

Group	Thickness of connective tissue septa, microns	
	Before injection of the MSC	One month after injection of the MSC
1	38.3 (38.5-44.8)	43.6 (23.6-55.2)
2	72.1 (67.8-77.5)	80.4 (73.9-90.1)
3	77.3 (70.1-87.2)	53.3 (49.4-57.2)

## Discussion

Group number one

There was no significant difference in septa thickness between experimental animals in group one at the start of the experiment and after one month (p = 0.39, Wilcoxon criteria). Dystrophic alterations were minor and did not occur in this group on both occasions. The number of double-core cells remained constant in this group throughout the trial (p = 1.0).

Group number two

There was no significant difference in septa thickness between experimental animals in group two at the start of the experiment and after one month (p = 0.09, Wilcoxon criteria). The dystrophic alterations were most apparent and did not change during the trial (p = 0.482). This group had a significantly higher number of double-core cells throughout the trial (p<0.001), which can be attributed to compensatory liver responses and strong hepatocyte proliferative activity during the illness.

Group number three

The thickness of fibrous septa in experimental animals in group three decreased significantly (p<0.001, Wilcoxon criteria) between the start of the experiment and after one month. At the start of the trial, all animals showed dystrophic alterations. One month later, MSCs were found in 20% of instances (p<0.001) but were weakly expressed and limited to the hepatic lobules' periphery. By the end of the trial, this group had considerably fewer double-core cells (p<0.001).

Thus, it may be concluded that administering autologous MSCs to rabbits with a single intraportal injection of 5x10^6^ cells per kg of body weight improved the pathohistological picture in experimental cirrhosis of the liver. Morphometric data confirms the positive effect of cell therapy on experimental liver cirrhosis, with a statistically significant decrease in connective tissue septa thickness (p<0.001, Wilcoxon criterion), number of bi-nucleated cells (p<0.001, Wilcoxon criterion), and severity of dystrophic changes in hepatocytes (p<0.001, Wilcoxon criterion) in the experimental group one month after the introduction of MSCs. One month after cell transplantation, there was a significant difference in connective tissue septa thickness (p<0.001, Mann-Whitney criterion), number of bi-nucleated cells (p<0.001, Mann-Whitney criterion), and severity of dystrophic changes in hepatocytes (p<0.001, Mann-Whitney criterion) between animal groups two and three.

Clinical studies on animal models, as well as recent human trials, have shown that mesenchymal stem cells can be used to treat liver cirrhosis and end-stage liver disease. Lu et al. (2023) and Liu at el. (2022) found from a meta-analysis of randomized control trials that mesenchymal stem cells are beneficial in treating liver cirrhosis in both acute and chronic stages [12,13]. Compared to standard therapy, mesenchymal stem cells improved liver function (MELD score, albumin levels, and coagulation function). Cao et al. (2020) identified the method by which MSCs counteract the consequences of liver fibrosis and cirrhosis [14]. It was noted that MSCs' ability to differentiate into hepatic cells, as well as their immunomodulatory capabilities and ability to produce trophic factors, contributed to their beneficial effects. This was corroborated by Wang and Chen (2022), who also said that combined treatment with MSCs had better outcomes [15]. Wang et al. (2024) found that the therapy is dose-dependent; however, greater dosages are not more effective than lower ones [16]. According to all of the research, adverse consequences are uncommon, and if they do occur, they are mild. There is a general consensus that additional study is needed on this subject so that suitable and safer ways may be pursued, thereby minimizing the likelihood of undesirable effects. There might be some limitations to this study. This study's possible weakness might be its limited sample size. A larger sample size may minimize the margin of error. We attempted to recreate identical settings in all experimental models, but there may still be confounding variables and complicated interactions between independent variables. It is also important to note that, because the model is animal-based, it is possible that a complete replication of the findings of this study will not be applicable in human studies due to the degree of genetic variability. A longer study duration may yield more accurate results.

## Conclusions

Transplanting autologous mesenchymal adipose tissue stem cells into rabbits with a single injection into the portal vein at a dose of 5x10^6^ cells per kg of body weight improves experimental liver cirrhosis.Morphometric data confirms cell therapy's therapeutic effect in experimental liver cirrhosis, with a significant decrease in connective tissue septa thickness (p<0.001, Wilcoxon criterion), number of bi-nucleated cells (p<0.001, Wilcoxon criterion), and severity of dystrophic changes in hepatocytes (p<0.001, Wilcoxon criterion) in the experimental group one month after the introduction of MSCs. One month after cell transplantation, there was a significant difference in the thickness of connective tissue septa (p<0.001, Mann-Whitney criterion), the number of bi-nucleated cells (p<0.001, Mann-Whitney criterion), and the severity of dystrophic changes in hepatocytes (p<0.001, Mann-Whitney criterion) between experimental groups two and three.
